# Bioimaging Innovations in Bionics and Biomechanics

**DOI:** 10.1155/2019/3651309

**Published:** 2019-10-14

**Authors:** Yuan-Chiao Lu, Ozan Erol, Santiago Orrego, Yao Wu, Li Zhao

**Affiliations:** ^1^Division of Diagnostic Imaging and Radiology, Children's National Medical Center, Washington, DC, USA; ^2^Hopkins Extreme Materials Institute, Johns Hopkins University, Baltimore, MD, USA; ^3^Department of Oral Health Sciences, Maurice H. Kornberg School of Dentistry, Temple University, Philadelphia, PA, USA; ^4^Department of Biomedical Engineering, George Washington University, Washington, DC, USA

## 1. Introduction

Recent advances in computational and bioimaging techniques have greatly enhanced the ability of engineers and scientists to better understand the dynamics of the human body and functions of living organisms. Specifically, progressive computational, mathematical, and physics-based approaches have helped researchers to develop sophisticated novel techniques that can solve the problems encountered in the fields of bionics and biomechanics with improved visualization of biological systems and design of new medical devices. These advances in imaging and visualization methods are helping to identify, classify, and quantify patterns in bionics and biomechanical investigations.

The theme of this Special Issue is to explore the state of the art of bionics and biomechanical research using bioimaging technology. The Special Issue consists of original contributions addressing challenges in understanding the mechanics and functions of biological systems and living organisms solved by innovative bioimaging methods. The scope of the Special Issue includes the bioimaging analysis in biomechanics, investigation of the functions of living organisms through imaging techniques, inventions of biomedical devices, and evaluation of surgical treatments. These original research studies provided the biomedical research community an insight into novel biomedical imaging techniques and algorithms on bionics and biomechanics.

## 2. Description of the Special Issue

This Special Issue accepted 6 papers out of 15 through careful review by editors and peer review, which led to an acceptance ratio of 40%. These 6 original research articles investigated the radiography system improvement, radiographic image analysis, medical image modeling, and imaging technique evaluation.

Y. Liu et al. proposed a novel data-driven decomposition model to decompose the conventional chest radiograph into both soft tissue and bone images and compared the results with virtual dual-energy subtraction (DES) imaging. The proposed approach markedly reduced the visibility of bony structures in chest radiographs and produced soft tissue and bone contrast similar to those produced by the actual DES system. Their work shows potential to enhance diagnosis of lung diseases.

Y.-H. Chang et al. constructed a finite element mandible model (cortical bone, cancellous bone, miniplate, and screws) from high-resolution computed tomography (CT) images to investigate the biomechanical structures of four common occlusion conditions after bilateral sagittal split osteotomy surgical treatment. They observed high stress on the miniplate for all four occlusion conditions, and the screws on the proximal segment near the bone gap experienced high stress. This platform provides more information on the biomechanics of mandible implantation.

W.-E. Hsu et al. compared 15 vertebral measurements on radiographic images of 18 patients with single-level vertebral compression fracture to access the degree of vertebral body height loss and kyphotic angle. The evaluation of these measurements could help to determine the probability of intravertebral clef, and this study could provide a reference for surgeons when using imaging modalities to access the degree of vertebral body collapse.

C.-W. Liao et al. developed a high-frame-rate intraoral periapical sensor with a senor imaging speed of up to 15 Hz for a 2.5D periapical radiography system, which could be used to capture images at different depths of an object. The developed sensor could be combined with tomosynthesis to obtain reconstructed slice images of different depths and has the potential for clinical dentistry applications.

K. Oberhofer et al. fit a generic musculoskeletal model of the lower limbs of an adult female subject to 3D body surface data of children with and without cerebral palsy. They compared the fitted lengths and volumes of six muscle-tendon structures with the subject-specific muscle-tendon lengths and volumes derived from magnetic resonance images. High accuracies were obtained in the fitted lower limbs in both study groups for 3D body surface data, but the accuracies of muscle volumes contained large variations.

S. Shimawaki et al. performed CT imaging on the fingers of 10 male adults gripping cylinders of three different diameters (10, 60, and 120 mm) and constructed 3D computational bone models based on these CT images to measure the flexion angle of each finger joint. Results showed that smaller cylinder diameters were associated with significant increases in the flexion angle of the all joints of four fingers. Consistent results were observed when comparing to the flexion angles of joints using other published methods.

